# A Review of the Neuroprotective Properties of Exosomes Derived from Stem Cells and Exosome-Coated Nanoparticles for Treating Neurodegenerative Diseases and Stroke

**DOI:** 10.3390/ijms26083915

**Published:** 2025-04-21

**Authors:** Yu-Ping Yang, Christopher J. B. Nicol, Ming-Chang Chiang

**Affiliations:** 1 Sylvester Comprehensive Cancer Center, Miller School of Medicine, University of Miami, Miami, FL 33136, USA; yyang22@med.miami.edu; 2 Department of Biochemistry and Molecular Biology, Miller School of Medicine, University of Miami, Miami, FL 33136, USA; 3 Departments of Pathology & Molecular Medicine and Biomedical & Molecular Sciences, and Cancer Biology and Genetics Division, Sinclair Cancer Research Institute, Queen’s University, Kingston, ON K7L 3N6, Canada; nicolc@queensu.ca; 4 Department of Life Science, College of Science and Engineering, Fu Jen Catholic University, New Taipei City 242, Taiwan

**Keywords:** neuroprotective, SC-Exos, exosome-coated NPs, neurodegenerative diseases, stroke

## Abstract

Neurological diseases, including neurodegenerative disorders and stroke, represent significant medical challenges due to their complexity and the limitations of current treatment approaches. This review explores the potential of stem cell (SC)-derived exosomes (Exos) as a transformative therapeutic strategy for these diseases. Exos, especially those derived from SCs, exhibit natural targeting ability, biocompatibility, and the capacity to cross the blood–brain barrier (BBB), making them ideal vehicles for drug delivery. This review provides an in-depth discussion of the properties and advantages of SC-Exos. It highlights their potential synergistic benefits in therapeutic approaches to treat neurological diseases. This article discusses the mechanisms of action of SC-Exos, highlighting their ability to target specific cells, modulate disease pathways, and provide controlled release of therapeutic agents. Applications in specific neurological disorders have been investigated, demonstrating the potential to improve outcomes in conditions such as Alzheimer’s Disease (AD), Parkinson’s Disease (PD), and stroke. Moreover, Exos-coated nanoparticles (NPs) combine the natural properties of Exos with the multifunctionality of NPs. This integration takes advantage of exosome membrane biocompatibility and targeting capabilities while preserving NPs’ beneficial features, such as drug loading and controlled release. As a result, Exos-coated NPs may enhance the precision, efficacy, and safety of therapeutic interventions. In conclusion, SC-Exos represent a promising and innovative approach to treating neurological diseases.

## 1. Introduction

### 1.1. Overview of Neurological Diseases

Neurological diseases encompass many disorders that affect the central and peripheral nervous systems [1]. These conditions can significantly impair quality of life and pose substantial challenges in diagnosis and treatment. Neurological disorders can be categorized based on various criteria, including the primary location affected, the type of dysfunction, or the underlying cause [2]. Some key categories include neurodegenerative diseases, which are characterized by the progressive loss of neuronal structure and function. An example of this condition is AD, which leads to memory loss and cognitive decline [3]. PD impacts motor function, leading to symptoms like tremors and stiffness [4]. Cerebrovascular Diseases involve disorders related to the blood vessels in the brain. Stroke occurs when the blood supply to the brain is interrupted, resulting in brain damage [5]. Treatment approaches for AD typically involve cholinesterase inhibitors and NMDA receptor antagonists to manage symptoms. Recent advancements include immunotherapy that targets amyloid beta plaques. However, some therapies have been associated with severe side effects, such as brain bleeds [6]. Dopaminergic medications for PD are the standard treatment. Innovations such as adaptive deep brain stimulation (DBS) have emerged, offering responsive modulation of brain activity and enhancing symptom management [7]. The acute management of a stroke includes thrombolytic therapy and mechanical thrombectomy [8].

Current treatments are typically limited to symptomatic relief and do not effectively halt or reverse disease progression. Treating neurological diseases involves overcoming several key challenges. The BBB is a highly selective barrier that protects the brain from harmful substances in the bloodstream. However, it also restricts the systemic delivery of therapeutic agents, making it difficult for them to cross [9]. The brain’s complexity requires targeted treatment due to its intricate morphology and localized neural damage [10]. Many current drugs have significant side effects because they cannot precisely target diseased brain regions. The brain has a limited ability to regenerate neurons, making recovery from neural damage particularly challenging [11]. Recent advances in biotechnology and nanotechnology offer promising new avenues for overcoming some of these challenges when treating neurological diseases [12,13], including the use of Exos-based therapies and NP-mediated drug delivery, which have shown significant potential [14].

### 1.2. Potential of Exos

Exos are small, membrane-bound vesicles secreted by cells that play a crucial role in cell-to-cell communication [15,16]. They can transfer proteins, lipids, and genetic material between cells, influencing various physiological and pathological processes. The unique properties of Exos, such as their biocompatibility, ability to cross the BBB, and intrinsic targeting capabilities, make them attractive candidates for drug delivery systems [17,18]. SCs can differentiate into various neural cell types and secrete Exos that carry neuroprotective and regenerative factors [19,20,21]. SC-Exos are particularly promising for treating neurological diseases due to their natural affinity for brain tissues and their content of bioactive molecules that support neural health and repair [19,22,23].

### 1.3. Integrating Exos and NPs

NPs are tiny particles, typically 1 to 100 nanometers, engineered to deliver therapeutic agents to specific cells or tissues [24]. They can be designed to improve drug solubility, protect therapeutic agents from degradation, and ensure controlled release. NPs have shown potential in improving the delivery of drugs across the BBB and ensuring targeted action within the brain [25]. Exos-coated NPs were created by merging the advantages of Exos with those of NPs. This hybrid approach leverages the natural targeting and communication abilities of Exos with the versatility and efficiency of NPs [26,27,28], creating a potent delivery system for neurological therapies.

### 1.4. Aim

This review aims to explore the potential of Exos in treating neurological diseases. It will cover Exos’ properties and therapeutic attributes, the advantages of their use in drug delivery systems, and how the combination of Exos and NPs can address current challenges in Neurotherapy. Furthermore, it will discuss their mechanisms of action, applications in the preclinical and clinical research of specific diseases, and future directions in this emerging field.

## 2. Exos

### 2.1. Definition of Exos

“Exos” is short for exosomes, an extracellular vesicle (EV) type. Exos are small, membrane-bound vesicles typically measuring 30–150 nm in diameter [29,30,31]. They originate from the endosomal system and are formed through the inward budding of multivesicular bodies (MVBs). Exos are released into the extracellular environment upon fusion of these MVBs with the plasma membrane. Exos play a crucial role in intercellular communication by transferring bioactive molecules, including proteins, lipids, mRNA, microRNA (miRNA), and other non-coding RNAs to recipient cells [32]. They are vital in various physiological processes, such as immune response, tissue regeneration, and neural communication. Additionally, Exos are implicated in several pathological conditions, including cancer, neurodegenerative diseases, and cardiovascular disorders [33]. The term “Exos” is often used interchangeably with “extracellular vesicles” (EVs). Still, it is important to note that Exos represent a specific class of EVs with unique biogenesis, size, and molecular composition characteristics. While EVs is a broad term that includes Exos, microvesicles (MVs), apoptotic bodies, and other types of vesicles, using “Exos” helps to highlight those vesicles that are derived from endosomes, as opposed to microvesicles, which bud directly from the plasma membrane. Exos are especially relevant in therapeutic applications, particularly in treating neurological diseases, regenerative medicine, and drug delivery [4]. This relevance is due to their ability to cross the BBB, their high stability, and their intrinsic targeting capabilities.

### 2.2. Sources of Exos

Exos differ significantly based on their cellular origin, which affects their composition, biological functions, and therapeutic applications [20,34]. A comparison of stem cell-derived exosomes (SC-Exos) with Exos from other donor cells regarding their characterization and functions can be seen below (Table 1, [32,35,36,37,38,39,40,41,42,43,44]). SC-Exos are particularly well-suited for regenerative and neuroprotective applications because they contain a unique cargo of growth factors, neurotrophic factors, and anti-inflammatory molecules [45]. In contrast, Exos from other donor cells exhibit functions specific to their cell type. These functions can be beneficial, such as neuron-derived Exos that support synaptic function, or detrimental, such as cancer-derived Exos that promote tumor progression. Due to their immunomodulatory, anti-apoptotic, and regenerative properties, SC-Exos hold superior therapeutic potential for treating neurodegenerative diseases and stroke [46,47].

### 2.3. Exos Extraction and Isolation Methods

Efficient and reliable isolation techniques are crucial to studying and utilizing Exos for therapeutic and diagnostic purposes [22,31,34]. The most common methods include Ultracentrifugation (UC), the gold standard for Exos isolation. It involves differential centrifugation at high speeds (approximately 100,000× *g*). Size Exclusion Chromatography (SEC) is a technique that separates Exos based on their size. This results in high purity while preserving their functionality. Polymer-based precipitation, such as ExoQuick, utilizes polyethylene glycol (PEG) to aggregate Exos and aid their precipitation. Ultrafiltration is a technique that uses membrane filters to separate Exos based on molecular weight, ensuring high recovery efficiency. Immunoaffinity capture involves targeting specific surface markers on Exos, such as CD9, CD63, and CD81, using antibody-coated magnetic beads to achieve high specificity.

### 2.4. Biological Properties of Exos

Exos have unique characteristics that make them valuable for therapeutic applications: they are biocompatible and exhibit low immunogenicity [41]. Because Exos are derived from natural cellular processes, they are generally well-tolerated in living organisms. Exos can traverse the BBB, making them suitable for delivering drugs and biomolecules to the central nervous system (CNS) [48]. The surface markers of Exos facilitate selective interactions with particular cells and tissues. Cargo delivery and controlled-release Exos naturally transport therapeutic biomolecules to target cells, promoting precision medicine [49]. Exos represent a powerful tool in regenerative medicine, drug delivery, and biomarker discovery.

### 2.5. Therapeutic Potential in Neurological Therapies

The therapeutic potential of SC-Exos lies in their unique properties and bioactive contents. SC-Exos may deliver neuroprotective proteins and RNAs that help to shield neurons from apoptosis and other forms of cellular stress [31,34,40]. SC-Exos may also modulate immune responses by carrying anti-inflammatory cytokines and other regulatory molecules, reducing the neuroinflammation associated with many neurological disorders [34]. In addition, the growth factors and signaling molecules within SC-Exos may stimulate the proliferation and differentiation of endogenous neural stem cells, aiding in tissue repair and regeneration [50]. Furthermore, SC-Exos may improve synaptic plasticity and support neuronal network remodeling, crucial for recovery in neurodegenerative conditions and brain injuries [34,51]. SC-Exos offer several advantages for neurological therapies [52,53,54]. Due to their biocompatibility, Exos derived from human cells are less likely to provoke immune responses than synthetic delivery systems. Their natural origin enables better integration and acceptance by neural tissues, promoting the targeted delivery of therapeutic agents. Exos possess the unique ability to cross the BBB, a critical challenge in treating neurological diseases. The bioactive molecules in SC-Exos are naturally optimized for neural health and repair.

### 2.6. Current Research and Developments

Recent research has focused on optimizing the production, isolation, and therapeutic application of SC-Exos [19,50,55]. Engineered Exos loaded with specific drugs, RNA molecules, and gene-editing tools are increasingly being used due to their ability to enhance therapeutic efficacy. An improved mechanistic understanding of how SC-Exos exert their effects has also facilitated their optimized use in various neurological conditions. Numerous animal studies have reported the potential of SC-Exos in models of neurodegenerative diseases and stroke, showing promising results regarding functional recovery and neuroprotection [34,56]. This comprehensive understanding of SC-Exos provides a foundation for their potential use in advanced therapies for neurological disorders, leveraging their natural properties and therapeutic potential.

## 3. Mechanisms of Action

### 3.1. Targeting and Uptake

Upon administration, the Exos travel through the bloodstream and cross the BBB using mechanisms such as receptor-mediated transcytosis [32,57]. The exosome surface proteins interact with receptors on the target cells, facilitating specific binding and uptake [58]. The mechanisms by which SC-Exos targets and is taken up by neural cells are intricate and multifaceted. Exos are naturally equipped with various surface proteins and ligands that can recognize and bind to specific receptors on target cells [59]. SC-Exos may leverage these surface molecules to home in on neural cells, expressing corresponding receptors [22,60]. For example, Exos may target neurons or glial cells by binding to cell surface markers like CD63, CD81, and L1CAM. In addition, upon binding to target cell receptors, Exos are internalized through endocytic pathways such as clathrin-mediated endocytosis, caveolin-mediated endocytosis, or micropinocytosis [61,62]. In some cases, phagocytic pathways can also be involved, especially in microglia, the brain’s resident immune cells. In specific scenarios, Exos may fuse directly with the plasma membrane of the recipient cell, releasing their cargo directly into the cytoplasm [58,63]. Specific membrane fusion proteins and lipid compositions of the exosome and target cell membranes facilitate this process.

### 3.2. Cargo Delivery and Release

The therapeutic agents, including drugs, RNA molecules, or proteins, are delivered to the site of action within the target cells [34]. This can lead to the modulation of cellular pathways, a reduction in pathological processes, and the promotion of cellular repair and regeneration. The delivery and release of therapeutic cargo from SC-Exos involves several critical steps [34], including Controlled Release—once in the cytoplasm, the cargo (which could include drugs, RNA molecules, or proteins) is released in a controlled manner [49]—and Nuclear Targeting—some Exos cargo needs to reach the cell nucleus for gene therapy applications [50,64].

### 3.3. Cellular and Molecular Effects

**Figure 1:**
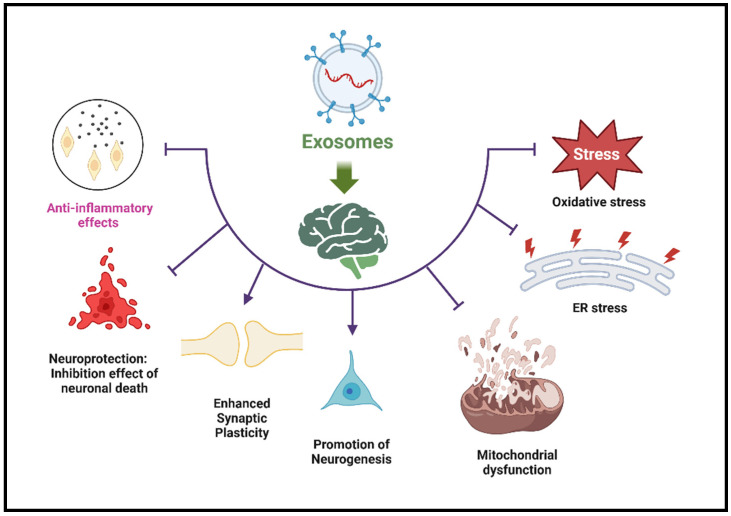
**Mechanisms of Exos’ effects on brain health.** This figure illustrates the cellular and molecular effects of Exos on brain health. It highlights beneficial anti-inflammatory effects that reduce neuroinflammation and neuroprotection that prevents neuronal death. Enhances synaptic plasticity—supports learning and memory. Promotes neurogenesis—encourages the formation of new neurons. Antioxidative stress—reduces ROS damage. Fights ER stress—reduces effects on protein folding and cellular homeostasis. Anti-mitochondrial dysfunction—reduces damage to energy metabolism.

The SC-Exos components may exert therapeutic effects by influencing gene expression, reducing inflammation, and promoting cell survival and growth [4,19]. The therapeutic effects of the cargo delivered by SC-Exos occur at multiple cellular and molecular levels. Therapeutic proteins delivered by SC-Exos can replace defective proteins, inhibit harmful enzymes, or stimulate protective pathways [34]. For example, delivering neurotrophic factors like BDNF (Brain-Derived Neurotrophic Factor) can promote neuronal survival and growth [40]. The anti-inflammatory targeting of SC-Exos includes cytokines and molecules that modulate the immune response, reducing neuroinflammation, a hallmark of many neurological diseases [34,40]. For example, visualization studies have shown that SC-Exos delivers anti-inflammatory agents (e.g., cytokines, small molecules) to brain regions affected by inflammation [31]. Reduction in toxicity can be achieved through various approaches [65]. Studies have shown that SC-Exos delivers targeted drugs precisely to diseased cells, reducing off-target effects and systemic toxicity. Similarly, therapeutic agents encapsulated within SC-Exos provide a protective barrier that prevents degradation and reduces toxic side effects. In addition, the natural biocompatibility of SC-Exos and their efficient clearance from the body minimizes long-term toxicity and immune reactions [34]. Exos’ cargo can support neuronal health by reducing oxidative stress, inhibiting apoptosis, and promoting cellular repair mechanisms [33,47,66,67,68,69,70]. Molecules such as growth factors and miRNAs in the Exos enhance neurogenesis and synaptic plasticity, facilitating recovery from neural damage. For example, SC-Exos delivery of neurotrophic factors (e.g., BDNF, GDNF) to support neuron survival, growth, and repair was reported [19,71]. Other studies showed SC-Exos delivering antioxidants that neutralize reactive oxygen species (ROS) and reduce oxidative stress in neuronal cells. SC-Exos can also promote the proliferation and differentiation of neural stem cells, contributing to the regeneration of damaged neural tissue [19,20,31]. Another study shows the role of SC-Exos in enhancing synaptic plasticity and improving both neural network function and cognitive outcomes [72]. Target cells internalizing SC-Exos via receptor-mediated endocytosis enable the intracellular delivery of therapeutic agents [73]. Other studies showed the controlled and sustained release of therapeutic cargo from SC-Exos within target cells, ensuring prolonged therapeutic effects [34,74]. The mechanisms of action by which SC-Exos modulates neuroinflammation, reduce toxicity, and promote neuroprotection highlight their multifaceted therapeutic potential in treating neurological diseases [75]. Mitochondrial dysfunction and endoplasmic reticulum (ER) stress are key characteristics of several health conditions, such as neurological diseases [76]. These conditions are often associated with impaired energy metabolism, increased oxidative stress, and cellular damage from mitochondrial failure and ER stress. As a result [77], using Exos for mitochondrial-targeted therapy to restore or enhance mitochondrial function has emerged as a promising strategy to alleviate the effects of these debilitating diseases. The relationship between Exos and ER stress pathways has garnered significant attention due to their roles in developing various human diseases and potential therapeutic applications [78]. In neurodegenerative diseases, ER stress, which involves misfolded proteins such as tau and α-synuclein, impacts the biogenesis and release of Exos. The interplay between exosome and ER stress pathways is a crucial factor in the progression of many diseases. The mechanisms by which Exos contribute to neuroprotection is summarized in Figure 1. The source and cargo of Exos influence their effects on brain health. Utilizing their therapeutic potential while reducing harmful effects presents a promising approach to treating neurodegenerative diseases.

### 3.4. Crosstalk with the Microenvironment

SC-Exos interact dynamically with the brain’s microenvironment [19,34,51,53]. Exos facilitate communication between different cell types in the brain, including neurons, astrocytes, and microglia. This intercellular communication is crucial for coordinating repair processes and maintaining homeostasis [79,80,81]. Components of SC-Exos can influence the ECM, promoting an environment conducive to tissue repair and regeneration. For example, Exos carry matrix metalloproteinases (MMPs) that can remodel the ECM, facilitating cell migration and tissue integration. By delivering anti-inflammatory agents and immunomodulatory molecules, SC-Exos can alter the behavior of immune cells in the brain, such as microglia and infiltrating lymphocytes, promoting a more regenerative and less destructive immune response [31]. SC-Exos are particularly effective at overcoming biological barriers that impede traditional drug delivery [82]. The BBB is a significant obstacle in treating neurological diseases. Exos naturally possess mechanisms to cross the BBB via transcytosis, making them ideal for delivering NPs loaded with therapeutic agents to the brain [32,44]. The dense and complex extracellular matrix in the brain can also hinder the movement of therapeutic agents. SC-Exos can navigate this environment more effectively due to their small size and natural composition [34,74].

## 4. Applications in Specific Neurological Diseases

The characteristics of SC-Exos make them suitable for various neurological applications [19,21,34,50,74].

### 4.1. AD

AD is characterized by the accumulation of amyloid beta plaques and tau tangles, leading to neuronal death, synaptic dysfunction, and cognitive decline [83,84,85,86]. The BBB poses a significant challenge in delivering therapeutic agents to the brain [87,88]. Therapeutic strategies with SC-Exos include Anti-Amyloid Therapies. SC-Exos can deliver siRNA or small molecules targeting amyloid precursor protein (APP) or beta-secretase, enzymes involved in amyloid beta production. This reduces plaque formation and promotes plaque clearance. In addition, the delivery of microRNAs or small interfering RNA (siRNA) can downregulate tau protein expression and prevent hyperphosphorylation, thereby reducing neurofibrillary tangles [89]. Exos from neural stem cells also contain neuroprotective factors such as BDNF, which can be delivered to support neuron survival and promote neurogenesis [31,81,90]. Finally, SC-Exos naturally carry anti-inflammatory cytokines that can modulate microglial activation and reduce neuroinflammation, a key component of AD pathology [91].

A summary of these papers on SC-Exos and their potential in AD are provided (Table 2). Adipose-derived Stem Cells (ASCs) Exos decreased Aβ deposition and apoptosis in neuronal cells derived from AD transgenic mice, highlighting their therapeutic effect in reducing neuronal damage [92]. Mesenchymal Stem Cells (MSCs)-derived Exos promoted neurogenesis and cognitive recovery in AD mouse models, supporting their potential to restore lost neuronal connections and cognitive functions [93]. MSC-derived Exos delivered intranasally exhibited immunomodulatory and neuroprotective effects in a 3xTg AD mouse model, effectively reducing neuroinflammation and preventing neuronal degeneration [94]. MSC-derived exosomal miR-223 regulated neuronal apoptosis by inhibiting pro-apoptotic pathways, highlighting a molecular mechanism for neuroprotection in AD [95]. MSC-derived Exos improved cognitive function and reduced Aβ aggregation in AD mouse models, showcasing their neuroprotective potential [96]. MSC-derived Exos enhanced autophagy and regulated insulin signaling by modulating the PI3K/Akt/mTOR pathway, suggesting a mechanism to alleviate AD pathology [97]. Neural Stem Cells (NSCs)-derived Exos promoted mitochondrial biogenesis and restored abnormal protein distribution in AD mouse models, indicating the potential for addressing mitochondrial dysfunction in AD [98]. NSC-derived Exos repaired the disrupted BBB in an in vitro model of AD, demonstrating their capacity to restore vascular integrity in neurodegenerative conditions [99].

In conclusion, SC-Exos show great potential for treating AD due to their immunomodulatory, anti-apoptotic, and neurodegenerative properties. They function through various mechanisms, including enhancing mitochondrial function, regulating autophagy, and repairing the BBB. These diverse abilities make them versatile tools for addressing the pathology of AD and improving cognitive function.

### 4.2. PD

PD involves the degeneration of dopaminergic neurons in the substantia nigra, leading to motor dysfunction [100,101]. Effective delivery of therapeutic agents to the brain is crucial for slowing disease progression and alleviating symptoms [102,103]. Therapeutic strategies with hNSC-Exos include encapsulating dopamine or dopamine agonists in SC-Exos, ensuring targeted delivery to dopaminergic neurons, enhancing therapeutic efficacy, and reducing peripheral side effects. The delivery of neuroprotective agents such as GDNF (Glial cell line-derived Neurotrophic Factor) also supports the survival of dopaminergic neurons [104]. Targeting the aggregation of alpha-synuclein with siRNA or small molecules delivered via SC-Exos to reduce toxic oligomer formation was also reported [105]. Finally, the delivery of genes encoding for enzymes involved in dopamine synthesis (e.g., tyrosine hydroxylase) restores dopamine levels in the brain [106].

These studies show that SC-Exos may reduce neuroinflammation, minimize neuronal apoptosis, enhance neurotrophic signaling, and improve metabolism in the diverse applications of Exos-based therapies for PD (Table 3). Exos derived from bone marrow-derived MSCs (BM-MSCs) containing the Gli1 protein were found to inhibit Sp1 signaling, reducing microglial activation and neuronal apoptosis [107]. These findings suggest a possible mechanism for reducing inflammation and cell death in PD. Exos derived from MSCs modulated cholesterol metabolism in neurons via the Wnt5a-LRP1 axis, improving cognitive function in a progressive PD model [108]. This study connects metabolic regulation to cognitive enhancements in PD. Exos derived from ASCs demonstrated therapeutic effects in a transgenic mouse model of PD by modulating neuroinflammatory and neurotrophic pathways [109]. This indicates their potential for enhancing motor function and alleviating PD pathology. Treatment with Ginkgolide A improved the functions of MSC-derived Exos, including their antioxidative and anti-inflammatory effects, in a 6-OHDA-induced PD cell model [110]. This study highlights the importance of pharmacologically enhancing Exos in treating PD. MSC-derived Exos delivered FTO-targeted siRNA, regulating m6A-dependent ATM mRNA to prevent the death of dopaminergic neurons [111]. This innovative approach combines Exos therapy with RNA interference to specifically target molecular mechanisms in PD. Human umbilical cord MSC-derived Exos loaded with BDNF enhance neuroregeneration and functional recovery in a PD model [112]. Neurotrophic factors significantly improve the therapeutic potential of Exos-based treatments.

### 4.3. Stroke

Stroke results in significant neuronal loss, neuroinflammation, and the disruption of the BBB [113,114]. The rapid and effective delivery of therapeutic agents is critical for minimizing damage and promoting recovery [115,116]. Therapeutic strategies with SC-Exos include the rapid delivery of neuroprotective agents such as antioxidants, anti-apoptotic molecules, and calcium channel blockers to protect neurons during the acute phase of injury [117,118]. Similarly, the delivery of growth factors such as VEGF (Vascular Endothelial Growth Factor) and BDNF stimulate the formation of new neurons and blood vessels, supporting tissue repair and functional recovery [119]. Also, the encapsulation of anti-inflammatory agents reduces secondary injury caused by neuroinflammation [120,121], as does the delivery of agents that promote the repair of the BBB, restore its integrity, and prevent further damage [122,123].

These studies highlight the potential of SC-Exos as a stroke treatment, emphasizing mechanisms like neuroprotection, anti-inflammation, and the reduction in oxidative stress (Table 4). IFN-γ stimulation improves the therapeutic potential of NSC-derived Exos, modulating immune responses and leading to better neuroprotection in an ischemic stroke model [22]. The miR-146a-5p found in Exos suppresses neuroinflammation by inhibiting the microglia’s IRAK1/TRAF6 signaling pathway, thereby reducing pro-inflammatory responses [124]. Exos miR-150-5p helps to reduce ischemia–reperfusion injury by targeting TLR5, which modulates inflammatory responses [125]. Exos lncRNA-ZFAS1 alleviates oxidative stress and inflammation by inhibiting miR-15a-5p, restoring homeostasis following a stroke [126]. Exos KLF4 reduces damage caused by ischemic stroke by modulating lncRNA-ZFAS1 to inhibit Drp1 methylation, thus preventing mitochondrial dysfunction [127]. Exos miR-193b-5p inhibits pyroptosis following an ischemic stroke by targeting AIM2, which helps to reduce inflammation-driven cell death [128]. NSC-derived Exos serve as nanocarriers for BDNF delivery, promoting neuronal repair and functional recovery after a stroke [40]. PD-L1- and HGF-decorated Exos enhance neuroplasticity by activating the STAT3-FOXO3 signaling pathway, which supports neuronal repair and functional recovery [129].

## 5. Exosome-Coated Nanoparticles (Exos-NPs)

### 5.1. Advantages of NPs in Neurological Disorders

Due to their ability to overcome significant therapeutic challenges, NPs have emerged as promising platforms for drug delivery, particularly in treating neurological diseases [13,76,130]. Some key advantages include improved drug solubility and bioavailability. NPs can encapsulate hydrophobic drugs, enhancing their solubility and extending their circulation time in the body [24]. Engineered NPs allow for sustained drug release, reducing dosing frequency and enhancing therapeutic efficacy [131]. Targeted drug delivery can be achieved by functionalizing NPs with targeting ligands, such as antibodies or peptides. This ensures selective delivery to diseased cells while minimizing off-target effects [132]. NPs can be designed to effectively cross the BBB, a significant challenge in neurological treatments [25]. This method utilizes the enhanced permeability and retention (EPR) effect in areas where the BBB is compromised, such as inflamed or tumorous regions [133]. Altering the surface of NPs with ligands that specifically bind to receptors on brain cells can improve their uptake [134]. Stimuli-responsive release NPs can be engineered to release drugs in response to specific stimuli, such as changes in pH, temperature, or enzymatic activity, ensuring drug activation occurs at the targeted site [135]. Intranasal delivery uses the olfactory and trigeminal nerve pathways to bypass the BBB, allowing direct access to the brain [136]. Magnetic targeting allows the precise delivery of magnetic NPs to specific brain regions using external magnetic fields, ensuring localized therapy [137].

### 5.2. Merging Exos and NPs: Strategies and Benefits

Exos combined with NPs create a highly effective delivery system that leverages both strengths [26,43]. Various methods, including direct adsorption, have been developed to coat, fuse, or merge Exos with NPs [138]. This method involves mixing Exos with NPs under controlled conditions, allowing for spontaneous adsorption while maintaining the integrity of the Exos. Layer-by-layer assembly involves alternating layers of oppositely charged molecules, including Exos, which result in a tunable coating with adjustable thickness and composition. Chemically modifying NPs can enhance covalent bonding, establishing strong covalent interactions with exosomal membrane proteins or lipids to ensure stable attachment [41]. This method utilizes charge-based attraction between Exos and NPs, creating a stable coating especially effective for charged nanoparticle surfaces. Bioorthogonal click chemistry involves engineered functional groups on Exos and NPs for precise, biocompatible conjugation, ensuring no interference with biological processes. These strategies showcase the complex yet advantageous interactions between Exos and NPs in developing advanced delivery systems. Exosomal coating enhances the properties and therapeutic potential of NPs through various mechanisms. Compared to synthetic coatings, it increases biocompatibility by reducing immunogenicity and toxicity, thereby improving the safety of therapies [139]. The surface proteins of Exos facilitate specific cell recognition and enhance uptake, enabling precise delivery to targeted brain regions [140]. Enhanced stability Exos protect NPs from premature degradation and clearance, prolonging their circulation time and increasing drug bioavailability. Exos naturally carry bioactive molecules such as neurotrophic factors, anti-inflammatory cytokines, and siRNA, which can enhance NPs-mediated therapies [37,138]. Exos provide better targeting, uptake, and biocompatibility, while synthetic NPs offer higher drug-loading capacity and controlled release (Table 5, [13,17,25,34,131,133,138,141,142,143,144]). Combining these natural and engineered systems in Exos-coated NPs creates an advanced platform for precision medicine, which is particularly beneficial for treating neurodegenerative diseases and stroke.

### 5.3. Properties of Exos-NPs

Exos-NPs have shown great potential in diagnosis and therapy (Table 6, [48,140,145,146,147,148,149]). These hybrid nanocarriers leverage the advantages of Exos, including their biocompatibility, ability to cross the BBB, and natural targeting capabilities, combined with the customizable properties of NPs to improve drug delivery. According to recent studies, Exos-NPs have become an emerging platform in nanomedicine due to their unique combination of synthetic NPs versatility and the natural targeting ability of Exos. Integrating Exos with NPs can enhance drug solubility, immune evasion, and targeted delivery, providing a powerful and versatile approach to treating complex diseases. Lopes et al. review bioengineered exosomal membrane-coated nanocarriers designed for neurodegenerative diseases and regenerative medicine [17]. Ribeiro et al. suggest using Exos-like liposomes to deliver natural compounds and RNA therapies to treat AD [150]. Liu et al. developed an Exos-coated gene-chem nanocomplex that acts as a nanoscavenger, effectively clearing alpha-synuclein aggregates and activating immune responses in PD [151]. In the context of ischemic stroke, Kim et al. developed magnetic extracellular nanovesicles derived from mesenchymal stem cells for targeted therapy [152]. Techniques such as surface coating, membrane fusion, and hybridization offer distinct advantages, particularly in drug delivery, gene therapy, and regenerative medicine. Incorporating Exos-NPs can enhance targeted drug delivery and improve the effectiveness of treatments. Exos-NP technologies are currently in preclinical stages, and research is ongoing to optimize their design, safety, and efficacy.

Mesenchymal stromal/stem cell-derived exosomes (MSC-Exos) are under clinical investigation in immunology, regenerative medicine, neurology, dermatology, endocrinology, and infectious diseases [153]. In one trial for chronic kidney disease, UCB-MSC-Exos administered intravenously at 100 g/kg produced significant improvements in estimated glomerular filtration rate, serum creatinine, blood urea, and urine albumin-to-creatinine ratio. In skin applications, ASC-Exos delivered by local injection (0.2 g twice daily) reduced melanin levels and enhanced skin texture, while an intravenous regimen (ExoFlo at 1–10 × 10^6^ MSCs/kg) in a COVID-19 trial improved survival and reduced inflammatory markers. Other trials report significant gains in pain reduction and disability scores in osteoarthritis, although some studies did not report clinical outcomes. Dosing strategies span microgram amounts to particle counts, with varying frequencies and routes (intravenous, local injection, inhalation). No clinical trial data on Exos-coated NPs were found.

### 5.4. The Translational Advances, Challenges, and Future Perspectives of Exos and Exos-NPs

Extracellular vesicles and their engineered derivatives, NPs, have emerged as promising tools in precision medicine, drug delivery, and regenerative therapies. Recent advances in Exos engineering, therapeutic cargo loading, and clinical applications highlight these biological NPs’ potential and challenges. Despite their promise, significant hurdles remain in optimizing their therapeutic efficacy, ensuring large-scale production, and establishing standardized data reporting frameworks for clinical comparability. Engineering and loading therapeutic Exos focuses on improving their natural capacity to carry bioactive molecules, such as proteins, RNAs, and small-molecule drugs. Tian et al. comprehensively review recent developments in Exos engineering for drug delivery [154]. Their study emphasizes various strategies for modifying Exos to improve targeting efficiency, including surface modifications with peptides and antibodies and genetic engineering to enhance endogenous cargo loading. These strategies have demonstrated improved therapeutic outcomes in preclinical neurodegeneration, cancer, and metabolic disease models. Similarly, Piffoux et al. discuss key exosome loading aspects, focusing on passive and active loading strategies [155]. Passive loading, which relies on diffusion-based methods, has limitations in achieving high cargo concentrations. In contrast, active loading techniques such as electroporation and sonication significantly enhance the efficiency of therapeutic payload delivery. However, these methods may also compromise the structural integrity of Exos, leading to functional alterations. A critical challenge remains to balance high-efficiency loading with preserving exosomal stability and bioactivity. Chen et al. further examine the encapsulation of therapeutic molecules within Exos [156]. They provide a systematic review of various techniques used to assess exosome loading and therapeutic effectiveness. Their findings underscore the importance of rigorous characterization methods, including high-resolution imaging and advanced omics-based profiling, to ensure that engineered Exos maintain their functional properties post-modification. The clinical translation of Exos-based therapeutics is a focus of intense research. Lee et al. critically review the clinical applications of Exos across multiple disease areas, including oncology, neurodegenerative disorders, and cardiovascular diseases [157]. They highlight several ongoing clinical trials where Exos formulations have shown promising therapeutic potential, particularly in targeted drug delivery. One example is using MSC-derived Exos to treat inflammatory diseases and regenerate tissue. However, Lee et al. also emphasize the variability in Exos production methods, which complicates regulatory approval and large-scale manufacturing. Further supporting this perspective, Perocheau et al. analyze the readiness of Exos-based therapies for clinical deployment [158]. They argue that while numerous preclinical studies have demonstrated efficacy, very few have progressed beyond early-stage trials due to concerns over batch-to-batch variability, immunogenicity, and stability during storage. These issues necessitate the development of standardized protocols for Exos isolation, purification, and quality control. Maumus et al. extensively explore the therapeutic potential of MSC-derived Exos, examining their regenerative capabilities in musculoskeletal and immune-related disorders [159]. Their study highlights the challenges in transitioning these vesicles from research models to clinical use, particularly regarding reproducibility and ensuring consistent therapeutic effects across different patient populations. Lener et al. proposed a foundational framework for addressing these translational barriers [160]. In their study, the international society for extracellular vesicles (ISEV) outlined key recommendations for using extracellular vesicles in clinical trials. Their position paper stresses the need for standardized manufacturing protocols and robust safety assessments to facilitate regulatory approval.

Data reporting and standardization pose critical challenges in this field, as the lack of a standardized framework limits the comparability of Exos-based studies. Piffoux et al. propose a structured framework for reporting exosome characteristics, cargo composition, and functional assays [155]. This initiative aims to improve reproducibility and ensure that data generated in preclinical models can be reliably translated into clinical applications. The study also highlights the importance of defining Exos purity metrics, as contamination with non-vesicular components can significantly alter therapeutic outcomes. Beyond technical standardization, Syn et al. explore the implications of Exos heterogeneity in cancer nanomedicine and immunotherapy [161]. Their research demonstrates that exosomal cargo composition varies depending on cell source, isolation method, and culture conditions, further complicating clinical standardization efforts. This variability underscores the need for a consensus on best practices for exosome production, characterization, and therapeutic evaluation. While Exos-based therapeutics hold immense potential, their clinical translation requires addressing several key bottlenecks. As Sharma et al. discussed, advances in engineering methods are improving Exos functionality, but large-scale manufacturing and quality control remain significant challenges [162]. Innovations in bioreactor-based production systems and cryopreservation techniques could solve these issues. Furthermore, Butreddy et al. discuss the broader implications of Exos-based biopharmaceuticals, including their use as delivery vehicles for nucleic acid-based therapies such as siRNA and mRNA [163]. The study highlights the need for more in-depth safety evaluations, particularly in assessing human subjects’ potential off-target effects and immune responses.

Engineering Exos-based NPs for therapeutic applications represents one of the most significant advancements in exosome research. This innovation aims to enhance therapeutic outcomes. These hybrid systems combine the biological advantages of Exos—such as biocompatibility, immune evasion, and natural targeting ability—with synthetic NPs tunability and enhanced stability. Several studies have provided insights into how these engineered Exos can be leveraged for clinical applications. For example, Chen et al. demonstrated the therapeutic potential of Exos-coated polydatin NPs in treating radiation-induced intestinal damage [146]. Their study highlighted the protective role of Exos functionalization, which improved NPs stability, enhanced cellular uptake, and increased therapeutic efficacy through anti-inflammatory and antioxidant mechanisms. This research underscores how Exos engineering can enhance drug bioavailability and mitigate side effects in oxidative stress and tissue injury conditions. Similarly, Hill et al. developed Exos-coated Prussian blue NPs for targeted glioblastoma therapy [149]. The study found that when used to coat Prussian blue NPs, tumor-derived Exos enabled the more precise targeting of glioblastoma cells while maintaining the ROS-scavenging properties of Prussian blue. This hybrid approach demonstrated increased therapeutic efficacy and reduced off-target toxicity, illustrating the potential of Exos-based targeting in oncology. In another study, Ye et al. investigated the role of Exos-based NPs in cancer immunotherapy [164]. Their work emphasized how engineered Exos could enhance immune responses by acting as antigen-presenting vesicles or delivering immune checkpoint inhibitors. The ability to modulate immune cell interactions through Exos engineering presents new opportunities for improving the efficacy of cancer immunotherapy. Furthermore, Yong et al. explored the use of tumor-derived Exos as drug carriers for chemotherapy [148]. Their research demonstrated that Exos-based NPs loaded with chemotherapeutic agents exhibited improved drug retention and enhanced tumor-targeting capabilities. The findings suggest that Exos-based carriers can help to overcome common limitations associated with conventional chemotherapy, such as systemic toxicity and poor drug penetration in solid tumors. Exos-based NPs are an innovative drug delivery and regenerative medicine solution, providing a promising alternative to synthetic nanoparticle systems. Recent research has shown significant advancements in Exos engineering, cargo loading, and targeting strategies, with applications in oncology, neurology, and inflammatory diseases. With ongoing advancements, Exos-engineered NPs have the potential to revolutionize precision medicine and targeted therapy in the years ahead.

## 6. Advantages of SC-Exos Therapies

SC-Exos offers superior targeting capabilities compared to traditional therapies [50,165]. Conventional drug delivery systems often suffer from non-specific distribution, leading to off-target effects and reduced therapeutic efficacy. Conventional systemic therapies often affect non-target tissues, leading to undesirable side effects. SC-Exos mitigates this issue through targeted delivery [34,50]. SC-Exos can address challenges related to drug resistance in neurological diseases. By facilitating efficient drug delivery to the brain, SC-Exos can ensure higher local concentrations of the therapeutic agent, potentially overcoming resistance mechanisms that limit drug efficacy [34,106]. Delivering siRNA or CRISPR-Cas9 components can also precisely silence or edit genes associated with drug resistance, providing a novel approach to overcoming therapeutic challenges [12]. Neurological diseases are often characterized by complex pathologies that necessitate a multifaceted approach to treatment. SC-Exos can deliver a combination of therapies that address various aspects of disease pathology, such as inflammation, oxidative stress, and synaptic dysfunction, offering a comprehensive treatment strategy [50,166]. The flexibility of SC-Exos allows for adapting the therapeutic payload to different stages of disease progression, from early intervention to advanced stages, ensuring continuous and effective disease management. By providing these significant advantages over traditional therapies, Exos-coated NPs represent a promising and innovative approach to treating neurological diseases [167], potentially transforming patient outcomes and paving the way for more effective and personalized medical interventions.

## 7. Challenges and Future Directions

### 7.1. Challenges

Overcoming biological barriers, such as the BBB and ECM, remains a significant challenge in the clinical translation of SC-Exos. Exos can cross the BBB, but efficiently delivering larger therapeutic cargoes to the brain remains challenging [57]. The complex and dense ECM in the brain can impede the movement of SC-Exos, limiting their distribution within target tissues and impacting their therapeutic efficacy [168]. Ensuring consistency and reproducibility in producing and characterizing SC-Exos is essential for clinical translation. In addition, the variability in exosome isolation methods can lead to differences in size, composition, and functionality, affecting the performance of the SC-Exos [169]. Standardized methods for characterizing SC-Exos are needed to accurately assess their physicochemical properties, stability, and cargo loading efficiency [170]. Modifying the surface properties of Exos and NPs (e.g., by incorporating targeting ligands or stealth coatings) may improve their stability, circulation time, and targeting specificity [145,171].

### 7.2. Immunogenicity and Safety

One of the key considerations in using SC-Exos is their immunogenicity and safety profile. SC-Exos tend to have lower immunogenicity than synthetic particles because they are derived from human cells. This reduces the risk of immune rejection and adverse inflammatory responses [21]. The use of biodegradable materials in SC-Exos and the natural clearance mechanisms of Exos contribute to a favorable safety profile, minimizing long-term accumulation and toxicity [74]. Addressing concerns related to immunogenicity and long-term safety is critical for the clinical success of SC-Exos. While Exos are generally considered immunologically inert, concerns remain about potential immune reactions to foreign components in the SC-Exos, such as synthetic polymers or therapeutic cargoes [172]. Long-term studies are needed to evaluate the potential accumulation and persistence of SC-Exos in the body and their impact on systemic health over time [50].

## 8. Conclusions

SC-Exos represent a cutting-edge and promising approach to revolutionizing the treatment of neurological diseases. By leveraging the natural targeting capabilities of Exos and the advanced delivery mechanisms of NPs, therapeutic agents can be delivered to the brain with unprecedented precision and efficacy, and give us the ability to explore the potential of SC-Exos in treating a variety of neurological diseases, including neurodegenerative diseases such as AD and PD, as well as stroke. The advantages of SC-Exos over conventional therapies are clear: enhanced targeting and delivery capabilities, reduced side effects and toxicity, multifunctionality, improved patient compliance, and versatility across various neurological diseases. These advantages stem from the unique properties of Exos, such as their ability to cross biological barriers, their biocompatibility and biodegradability, and their potential for customized therapeutic approaches. However, several challenges must be addressed to fully realize the clinical potential of SC-Exos. Overcoming biological barriers, ensuring standardization and quality control, addressing immunogenicity and long-term safety issues, and advancing delivery strategies are key areas for future research. In conclusion, SC-Exos offers a promising avenue for advancement in neurotherapeutics, potentially improving outcomes in patients affected by and treated for these diseases.

## Figures and Tables

**Table 1:** **Comparison of stem cell (SC)- derived exosomes (SC-Exos) and Exos from other donor cells.** Exos differ significantly based on their cellular origin, which affects their composition, biological functions, and therapeutic applications.

Feature	SC-Exos	Exos from Other Donor Cells	Study
Origin	Derived from mesenchymal stem cells (MSCs), neural stem cells (NSCs), embryonic stem cells (ESCs), and induced pluripotent stem cells (iPSCs).	Derived from neurons, glial cells, immune cells (e.g., macrophages, dendritic cells), cancer cells, fibroblasts, and endothelial cells.	[35,38]
Size and Morphology	Typically 30–150 nm, cup-shaped, enclosed in a lipid bilayer.	Similar to the size range (30–150 nm), morphology varies based on donor cell type.	[35]
Key Cargo Components	Enriched in growth factors (VEGF, TGF-β, IGF-1), anti-inflammatory cytokines (IL-10, TGF-β), neurotrophic factors (BDNF, NGF), and regenerative miRNAs (miR-21, miR-124, miR-146a).	Varies by cell type: neuronal Exos contain neurotransmitters and synaptic proteins; immune cell Exos carry cytokines; cancer cell Exos transport oncogenic proteins and miRNAs.	[40,41,42]
Main Functions	Tissue repair, immunomodulation, anti-inflammation, neuroprotection, angiogenesis, and cell survival promotion.	Cell-specific communication, immune regulation, neuroinflammation, tumor progression, or metastasis (depending on donor cell type).	[36,39]
Therapeutic Potential	Regenerative medicine, neurodegenerative disease therapy, stroke recovery, and drug delivery.	It can have pro-inflammatory, neurotoxic, or disease-promoting roles (e.g., in cancer and neurodegeneration) but also beneficial effects depending on the source.	[37,43]
BBB Penetration	High—SC-Exos naturally cross the BBB and promote neuroprotection.	Varies—some cell-derived Exos (e.g., from neurons) can cross the BBB, while others (e.g., fibroblast Exos) have limited BBB penetration.	[32,44]

**Table 2:** **SC-Exos and AD.** Exos derived from SCs (including ASCs, MSCs, and NSCs) show promising therapeutic effects in AD by reducing Aβ levels, enhancing autophagy, promoting mitochondrial biogenesis, increasing neurogenesis, and modulating the immune response.

Exosome Source	Model	Key Findings	Mechanisms	References
ASCs	Neuronal cells from transgenic AD mice	Decreased Aβ pathology and neuronal apoptosis.	Inhibited apoptosis in neuronal cells and reduced Aβ deposition.	[92]
MSCs	AD mouse model	Enhanced neurogenesis and cognitive recovery.	Promoted neurogenesis and increased neuronal connections.	[93]
NSCs	5 × FAD AD model	Restored blood–brain barrier integrity.	Exos repaired disrupted BBB.	[99]
MSCs	3 × Tg AD mouse model	Delivered immunomodulatory and neuroprotective effects.	Modulated immune response and protected neurons from degeneration.	[94]
MSCs	Neuronal cells (in vitro)	Regulated neuronal apoptosis.	Exosomal miR-223 inhibited pro-apoptotic pathways.	[95]
MSCs	AD mouse model	Improved cognitive deficits and reduced Aβ aggregation.	Reduced Aβ aggregation in the brain.	[96]
MSCs	Molecular pathways analysis	Enhanced autophagy and insulin signaling.	Modulation of PI3K/Akt/mTOR pathway to regulate autophagy and insulin signaling.	[97]
NSCs	AD mouse model	Promoted mitochondrial biogenesis and normalized protein distribution.	Activated mitochondrial biogenesis pathways and restored abnormal protein distributions.	[98]

**Table 3:** **SC-Exos and PD.** These articles explore the role of SC-Exos in providing neuroprotection in PD.

Exosome Source	Model	Key Findings	Mechanisms	References
BM-MSCs	Mouse PD model, in vitro	Gli1-containing Exos inhibited Sp1 signaling, reducing microglial activation and neuronal apoptosis.	Direct inhibition of Sp1 signaling, reducing microglial activation and neuronal apoptosis.	[107]
MSCs	Progressive PD model	Exos modulated neuron cholesterol metabolism via Wnt5a-LRP1, alleviating cognitive impairment.	Modulation of neuron cholesterol metabolism via the Wnt5a-LRP1 axis.	[108]
ASCs	Transgenic mouse PD model	Exos improved neuroprotection, reduced PD pathology, and enhanced motor function.	Modulation of neuroinflammatory pathways and enhanced neurotrophic signaling.	[109]
MSCs with Ginkgolide A treatment	6-OHDA-induced PD cell model	Ginkgolide A enhanced Exos neuroprotection and reduced neurotoxicity.	Enhancement of Exos pleiotropic effects, including antioxidative and anti-inflammatory actions.	[110]
MSCs with siRNA delivery	PD model	Exos targeting FTO via m6A-dependent ATM regulation alleviated dopaminergic neuronal death.	Regulation of m6A-dependent ATM mRNA through FTO-targeted siRNA delivery, reducing neuronal death.	[111]
Umbilical cord MSCs (BDNF-loaded)	PD model	BDNF-loaded Exos provided enhanced neuroprotection and functional recovery.	Delivery of BDNF to promote neurodegeneration.	[112]

**Table 4:** **SC-Exos and stroke.** These studies indicate advancements in the application of SC-Exos for treating ischemic stroke.

Exosome Source	Model	Key Findings	Mechanisms	References
NSCs	Mouse ischemic stroke model	IFN-γ-stimulated NSC-derived Exos enhance stroke recovery	Modulation of therapeutic capacity via stimulation	[22]
Umbilical Cord MSCs (UC-MSCs)	Mouse ischemic stroke model	miR-146a-5p reduces neuroinflammation by suppressing IRAK1/TRAF6 signaling	Modulation of inflammatory pathways	[124]
BM-MSCs	Rat cerebral ischemia/reperfusion model	Exos miR-150-5p targets TLR5 to reduce ischemia/ reperfusion injury	Suppression of TLR5-related pathways	[125]
BM-MSCs	Rat ischemic stroke model	Exos lncRNA ZFAS1 alleviates oxidative stress by inhibiting miR-15a-5p	Antioxidative and anti-inflammatory signaling	[126]
BM-MSCs	Mouse ischemic stroke model	Exos KLF4 inhibits m6A modification of Drp1 via lncRNA-ZFAS1, alleviating stroke injury	Modulation of epi transcriptomic pathways	[127]
BM-MSCs	Mouse ischemic stroke model	miR-193b-5p reduces pyroptosis by targeting AIM2, improving outcomes after ischemic stroke	Inhibition of AIM2-related pyroptosis	[128]
NSCs	Rat ischemic stroke model	Exos used as BDNF carriers improved outcomes in ischemic stroke rats	Delivery of BDNF for neuroprotection	[40]
MSCs	Mouse ischemic stroke model	PD-L1-HGF-decorated Exos enhance neuroplasticity via STAT3-FOXO3 signaling	Promotion of neuroplasticity pathways	[129]

**Table 5:** **Comparison of targeting and internalization mechanisms: Exos vs. NPs.** Understanding the differences in targeting and internalization mechanisms between Exos and synthetic NPs is crucial. Exos-coated NPs present a significant advantage in drug delivery and neuroprotection.

Feature	Exos	Synthetic NPs	Study
Targeting Mechanism	Intrinsic biological targeting—Exos naturally recognize and bind to recipient cells via ligand–receptor interactions, integrins, tetraspanins (CD9, CD63, CD81), and adhesion molecules.	Passive or active targeting—NPs rely on enhanced permeability and retention (EPR) effect for passive uptake or require chemical modifications (e.g., PEGylation, antibody conjugation, ligand attachment) for active targeting.	[17,133]
Cell-Specific Uptake	Highly cell-selective—Exos are preferentially taken up by cells from their parent tissue due to their surface proteins and homing signals.	Less cell-selective—NPs uptake depends on size, shape, surface charge, and ligand modifications. Requires functionalization for specific targeting.	[142,143]
Internalization Pathways	Internalized via clathrin-mediated endocytosis, caveolin-mediated endocytosis, micropinocytosis, and direct membrane fusion. Efficiently trafficked into recipient cells for cargo release.	Primarily taken up via endocytosis (clathrin/caveolin-dependent or independent). Often trapped in endosomes/lysosomes, leading to degradation before reaching the target site.	[141,144]
BBB Penetration	High—Exos naturally cross the BBB through transcytosis and receptor-mediated uptake (e.g., LRP1, integrins, and tetraspanins facilitate BBB transport).	Low—Most NPs require chemical modifications (e.g., PEGylation, ligand conjugation) or external forces (e.g., magnetic or ultrasound stimulation) to cross the BBB.	[25,34]
Immune Evasion	Excellent—Exos exhibit immune tolerance due to their biological origin, reducing immune clearance.	Variable—NPs are often recognized as foreign by the immune system. Surface modifications (e.g., PEGylation) are required to improve biocompatibility and prolong circulation.	[13,138]
Therapeutic Cargo Protection	High—Exosomal membranes protect encapsulated proteins, RNAs, and lipids from degradation.	Moderate—NPs protect cargo but may face aggregation, opsonization, or premature degradation in biological fluids.	[34,131]

**Table 6:** **Biomedical applications of exosome-coated nanoparticles (Exos-NPs) for diagnosis and therapy.** Exos-NPs have gained significant attention in biomedical research for their multifaceted capabilities in both diagnosis and therapy. By harnessing the intrinsic properties of Exos—such as biocompatibility, low immunogenicity, and natural targeting ability—Exo-NPs provide an advanced platform that enhances the therapeutic efficacy and diagnostic precision of conventional NPs.

Nanoparticle Type/Hybrid	Application/Target Disease	Key Findings	Study
Exos-coated polydatin NPs	Radiation-induced intestinal injury	Enhanced antioxidant and anti-inflammatory effects; improved repair of intestinal damage.	[146]
Hybrid Exos—organic/inorganic NPs	Broad (cancer, neurodegenerative disease, imaging)	Exos-NPs hybrids improve targeting, bioavailability, and diagnostic accuracy.	[145]
Biocompatible NPs encapsulated in Exos	Cancer theranostics	Efficient encapsulation and delivery; improved imaging and therapeutic efficacy.	[147]
Exos membrane-coated nanosystems	Cancer diagnosis and therapy	Exos membranes improve immune evasion, targeting specificity, and drug delivery efficiency.	[140]
Tumor-derived Exos NPs	Chemotherapy for cancer	Tumor Exos as carriers enhance drug delivery to tumor sites; improved therapeutic outcomes.	[148]
Exos-coated Prussian Blue NPs	Glioblastoma	Targeted accumulation in Glioblastoma; reduced toxicity; significant therapeutic effect.	[149]
Exos as lipid-based NPs	Drug delivery and diagnostics	Highlights Exos as “natural lipid NPs”; emphasizes their role in targeted, biocompatible delivery systems.	[48]

## Data Availability

Data will be made available on request.
